# Demonstration and Suggestion on the Communication Efficiency of New Media of Environmental Education Based on Ideological and Political Education

**DOI:** 10.3390/ijerph20021569

**Published:** 2023-01-15

**Authors:** Huiyu Ren, Liang Zhao

**Affiliations:** 1 School of Marxism, Wenzhou University, Wenzhou 325035, China; 2 School of Tourism, Hubei University, Wuhan 430062, China

**Keywords:** ideological and political education, environmental protection education, new media, spread efficiency

## Abstract

With the rapid development of the economy, we are facing more and more problems, and the construction of ecological civilization has become the focus of our national concern. With the rapid development of network technology, the immediacy of the new media and the huge audience have brought new development trends to the dissemination of environmental information. The number of new environmental media is increasing, but there are still some problems, such as formality, rigid content and lack of innovation, which make it difficult to achieve better communication effects. However, the research on new environmental media is still in its infancy, and there is not yet a set of targeted and specialized new media evaluation systems. Based on the social function of new environmental news media and the social responsibility of media as the entry point, the article establishes a set of index systems to measure the efficiency of environmental news dissemination and proposes corresponding improvement measures accordingly. The results of the study show that the best use of environmental education publicity is at 81.3%. In terms of cognitive efficiency and attitudinal efficacy, the scores of environmental education weibo public numbers were not high, at 60.7% and 71.5%, respectively. From the perspective of ideological and political education, the environmental protection class of WeChat plays a good role in attracting the attention of college students, and can provide ideological and political education to them and improve their ideological awareness. In terms of cognition, new media is responsible for conveying environmental knowledge and concepts to college students, so the development of new media centers on environmental information, and the content directly affects the cognitive level of college students, fully reflecting the importance of cognitive efficacy in new media of environmental education. On attitude efficacy, only one indicator is set for identity shaping, which has the highest score, but the lowest is 4.0, showing that the public number is still not obvious enough in terms of identifying with college student groups, influencing college students’ emotions and attitudes. Based on this, this paper points out the problems of the current communication efficiency of environmental education new media through the analysis of the evaluation results and puts forward suggestions to improve its communication efficiency in this regard.

## 1. Introduction

Xi Jinping, at the summit of world leaders, issued an important speech on the natural environment, “Jointly Building a Community of Human and Natural Life”, which said: “The threat of climate change to mankind is real, severe and long-term” [1]. The Fifth Plenary Session of the 19th Central Committee clearly set out the new goals and new missions of “Building a better China in all respects” and “Realizing new development of ecological civilization” in the 14th Five-Year Plan [2]. Since humans entered the industrial revolution, the balance of the Earth’s ecosystem has been gradually broken, and the deep contradiction between humans and nature has become more and more prominent. Problems such as land desertification, climate change, air pollution, and water pollution are becoming more and more serious, which not only affect the sustainable development of the world economy, but also seriously threaten the survival of mankind [3]. To solve the contradiction between man and nature, we must protect the ecology and promote the harmonious development of man and nature as we protect our own eyes. We must improve the ecological and cultural quality of the whole people, form a good ecological and cultural atmosphere in the whole society, and build an ecological civilization cultivation system [4]. To really make ecological civilization construction this way, it is not enough to focus only on the institutional level, as it must also be implemented ideologically [5]. The dissemination of ecological civilization is a proven way, which requires raising the ecological civilization awareness of citizens throughout society so that they can play the role of participants, builders, and supervisors in the construction. General Secretary Xi Jinping pointed out that it is necessary to strengthen the propaganda and education of ecological civilization, enhance the awareness of conservation, environmental awareness, and ecological awareness of all people, and create a good atmosphere for the protection of the ecological environment [6]. For the publicity and education of ecological civilization to be done well, the whole society should actively participate to form a good ecological civilization culture and promote the construction of an ecological civilization. The new media, on the other hand, plays a pivotal role in ecological civilization with its dissemination rate and breadth as a new way of ecological civilization [7,8]. In the Notice of the National Environmental Publicity and Education Action Plan (2011–2015), the state has developed five “environmental education series projects”, three of which are related to the Internet and new media platforms. The State Council’s “Guiding Opinions on Actively Promoting “Internet+” Action” mentions the concept of “Internet+ Green Ecology” and emphasizes the use of network technology to develop “Internet+”. New media plays a pivotal role in the dissemination of an ecological civilization, and its emergence is mainly reflected in various aspects such as government departments, non-governmental organizations, and media. Second, at this stage, new media have become the main way and means for the general public to receive information [9,10,11]. New media have provided a broader platform for the public, society, and enterprises to jointly participate in the construction of ecological civilization for communication and interaction. The large audience of new media and its impact on the way human society communicates have been noticed by various environmental media and groups, and the emergence of new environmental media has greatly promoted the popularity of ecological civilization ideas [12]. At the same time, the advantages and disadvantages of environmental new media communication effectiveness, the smoothness of communication channels, and the effectiveness of communication have also attracted widespread attention in the industry. Therefore, the construction of a more systematic, targeted, and referenceable evaluation index system for the communication effectiveness of environmental new media is important for the improvement of the communication efficiency of environmental new media, and can enhance public awareness of environmental protection, thus promoting the spread of ecological education. It can also enhance public awareness of environmental protection and thus promote the dissemination of ecological civilization.

In summary, evaluating the communication effectiveness of environmental new media from the perspective of political education can take political education as an orientation, so that the communication efficiency of environmental new media can be better utilized to make the propagation of ecological civilization more efficient, promote ecological and environmental knowledge, enhance college students’ environmental awareness, and influence their behavior, thus giving play to their unique social functions. Based on the above findings, this paper analyzes the effectiveness of environmental education new media communication in terms of contact effectiveness, cognitive effectiveness, and attitudinal effectiveness, and provides some theoretical support for better thinking and political education for college students.

## 2. Research Hypothesis

### 2.1. Contact Effectiveness and New Media Communication of Environmental Protection Education

Gonzalez (2020) pointed out in their research that media education is of great significance in enhancing citizens’ awareness of political participation and enhancing their ability to participate in politics [13]. Today, when information exchange increasingly relies on social media, people should critically access and analyze information, which is the cornerstone of their participation in social affairs and an important force of citizen participatory democracy [14]. The news contact of social media has a direct role in promoting the online political participation of young people, and the political efficacy plays the role of intermediary [15]. The news contact of young people’s social media can indirectly promote their political participation, thus enhancing their political effectiveness [16]. The development and application of the network make the political participation of young people more convenient and offers more opportunities. However, the generation of political participation depends not only on the cost and channels of participation, but also on the level of personal awareness [17]. The survey results show that, in today’s diversified media environment, the positive impact of social media on the political efficacy of the youth interviewed, although slightly worse than that of ordinary newspapers and magazines, is far greater than that of television and portal websites, indicating that social media has an increasing impact on youth political participation [18,19,20]. The political effectiveness of social media’s news dissemination is a key factor affecting the degree of the youth’s political participation [21]. Na, Xue-Xia, and Jia (2019) also believed in the research that the news contact of traditional media has an obvious positive effect on the political discussion in private occasions and the expression on the Internet [22]. However, this is not like the logic line set by Western scholars, which has a certain impact on the public’s participation, that is, the traditional media has not played any role in the public’s information dissemination [23]. This shows that, in the case of China, news participation will have a positive effect on participation behaviors that require relatively simple resources, such as daily discussions and online participation, but it has no significant impact on participation that requires more resources, such as putting forward opinions to government agencies and news media [24,25,26]. On the other hand, there is still debate about the impact of online media contacts on political participation [27]. Ren (2010) proposed in his research that, at the media contact level, the involvement of traditional media has had a significant impact on political participation, which shows that new media are increasingly prominent in the modern society. However, its influence cannot be underestimated in political life [28]. On the relationship between media contact and political efficacy, through multiple regression analysis, only TV media has a significant impact on political efficacy [29]. As far as specific political events are concerned, traditional media has a significant positive relationship with internal political efficacy, but does not have a stable impact on external efficacy [30]. Liu (2009) found in their research that the location and device of network contact have a significant impact on the richness and creativity of network content [31]. This further explains that we should comprehensively examine the deeper and more complex network content production form from the aspects of production content, form, and corresponding network self efficiency [32]. The openness, convenience, and freedom of Internet technology enable everyone to engage in content production [33]. In the context of new media technology, the production of online content is considered to be an important factor affecting democratic issues and social equity [34]. The research results show that parents’ socio-economic status and media contact status have a great impact on the generation of online content, especially in the early stage of new media technology communication, and the use of channels, tools, and costs of media contact status are the main factors that cause the difference in online content products [35,36,37]. Based on the above analysis, this paper proposes the following assumption:

**Hypothesis** **1.** *Contact efficiency can improve the communication effect of new media of environmental protection education.*

### 2.2. Cognitive Effectiveness and New Media Communication of Environmental Protection Education

Lu (2007) said in their research that cognition is an important concept in psychology [38]. It is the process of perception, judgment, reasoning, and thinking construction, and is the basis for people to make decisions and actions [39]. With the enhancement of enterprises’ awareness of environmental protection, enterprises’ understanding and grasp of green innovation, and under the integration of internal and external resources, they implement the “deep green” strategy [40,41]. First of all, the managers of the opportunity-oriented environmental awareness enterprises increase their R&D investment to develop their own green products, so as to effectively differentiate themselves from other enterprises and form different competitive advantages. Secondly, through technological innovation, environmental awareness managers master key environmental technologies and stay ahead in technology, so as to gain industry recognition, establish industry models, and improve the company’s environmental image [42]. Finally, environmental awareness managers can build a complete environmental management system to adapt to the environment, thus forming a positive response to environmental change [43]. By learning environmental protection knowledge and skills for internal personnel of the enterprise, environmental protection knowledge can be transformed into technology, thus improving the ability of the entire organization to “learn about environmental protection”, actively reflecting on its own environmental development, and implementing green innovation strategies to bring sustainable value to the company [44]. Chen (2017) pointed out that in terms of new media contacts and environmental awareness level of concern, respondents paid more attention to air pollution issues and were more interested in hot events related to haze [45]. In terms of cognition, the respondents have a high sense of trust in most of the online information about haze, but their awareness in reality is not ideal. In terms of attitude, the respondents believed that the government and the company played an important role in controlling air pollution, but they were obviously dissatisfied with the company’s performance. On the expectation level, the respondents disagreed on the time to improve the air quality and believed that measures could be taken to improve the work [46,47]. After a more in-depth analysis of the use of online news, we found that the more contact with or use of online news, the better the performance in the dimensions of attention, cognition, attitude, behavior, and expectation [48,49,50]. Cao (2014) believed in his research that, in recent years, China’s tourism industry has developed rapidly, driving the rapid development of China’s economy, but also causing a series of environmental problems [51]. For example, tourists littering, spitting, and destroying cultural relics and historic sites during tourism not only affect the image of the scenic spot, but also cause irreparable ecological problems in the scenic spot [52]. The main cause of tourism environmental problems is that tourists fail to consciously follow the social norms of tourism environmental protection [53]. Social norms refer to the code of conduct of people in social behavior, which is generated for the common needs and habits of society in social interaction, or jointly formulated and implemented by people. Its essence is the reflection of social relations and the concrete expression of social relations [54]. Individual behavior can be recognized, and recognized by the society, if it is consistent with social norms. Once individual behavior deviates from the social standard, it will be denied and condemned by the society [55]. In daily activities, groups that follow social norms can regulate their own behavior according to the established behavior, while in tourism activities, they consciously follow the tourism behavior norms of the scenic spot and consciously maintain the environment of the scenic spot [56,57]. Based on the above analysis, this paper proposes the following assumption:

**Hypothesis** **2.** *Cognitive efficacy can improve the communication effect of new media of environmental protection education.*

### 2.3. Attitude Effectiveness and New Media Communication of Environmental Protection Education

Cen (2001) believed that residents’ satisfaction with environmental work is an important indicator to measure the government’s environmental quality [58]. The public’s evaluation of environmental protection work is the result of the comprehensive influence of subjective understanding, objective conditions, personal factors, and macro factors. Environmental work satisfaction is an important indicator to measure a country’s credit. The higher the satisfaction of environmental work, the better the actual effect of the government on environmental protection work [59,60]. The material basis, institutional environment, organizational basis, and other factors of government environmental construction can provide a material and social environment for cultivating national environmental awareness [61]. Therefore, the higher the degree of social trust is, the more people tend to care about the ecological environment of the community and have a positive awareness of environmental protection. This requires people to constantly improve the public’s confidence in the environment through media publicity, system guarantee, education, and guidance, so as to consolidate and improve the degree of public participation in environmental protection [62,63]. However, it must be recognized that the specific mechanism of institutional trust depends on the interaction between institutions and citizens [64]. This means that people with higher system trust usually regard the system as a means to obtain and protect their own interests, based on pragmatism [65]. Therefore, the guiding role of civil system trust in public life in China is relatively weak, and it is difficult to effectively transform the positive attitude of citizens towards environmental protection through normative guidance [66]. Gao, Zheng, and Yan (2015) also mentioned in the study that the active support and participation of all sectors of society are necessary to achieve sustainable urban development [67]. Environmental protection is an active action taken by environmental actors to reduce or eliminate environmental pollution and reduce environmental burden under the impetus of environmental protection awareness [68]. A positive environmental attitude is a prerequisite for a citizen to protect the environment. To cultivate people’s awareness of environmental protection in their daily life, we must make the public aware of our obligations to protect the environment. Only in this way can citizens have the will to make some personal sacrifices to protect the environment, and will not blindly rely on the government, but actively carry out environmental protection activities in daily life [69,70]. The higher the public’s awareness of the importance of environmental protection, the stronger their awareness of environmental protection, and the stronger their environmental protection behavior in daily life [71]. Tian (2015) pointed out that people’s concern about the environment was related to the environmental problems they encountered. When facing water resource problems, people with stronger environmental awareness are more likely to take measures. People with negative views on environmental protection often choose to stand by and watch [72]. Residents’ water-saving actions are a special environmental protection behavior, which is embedded in personal social life. Its own socio-economic factors have a certain impact on it, which is the structural reason [73]. The study found that the impact of individual gender, age, education background, income, and other factors on the conservation-oriented environment, to some extent, proved the individual’s structural cause theory or structural constraint theory [74]. At the same time, in the specific behavior of environmental protection, the individual’s environmental protection behavior also shows the specific environmental behavior related to the individual’s corresponding cognition, knowledge, and attitude. The cognitive attitude model of individual environmental behavior can reflect the influence mechanism of individual environmental behavior. The environmental awareness and knowledge acquisition of participants and the formation of an environmental protection attitude will have a positive impact [75]. Therefore, to promote people’s participation in environmental protection, we must strengthen the popularization of environmental protection awareness and knowledge in actual social life and cultivate and enhance people’s environmental protection awareness through various forms of publicity and education [76]. Based on the above analysis, this paper proposes the following assumption:

**Hypothesis** **3.** *Attitude effect can improve the communication effect of environmental protection education new media.*

## 3. Research Design

### 3.1. Research Methods

This thesis takes the communication efficacy of environmental new media as an entry point from the perspective of Civic Education and establishes a corresponding communication efficacy assessment system. Then, the communication efficacy of green education-based new media is analyzed from three perspectives: contact efficacy, cognitive efficacy, and attitude efficacy. Secondly, the validity of this paper is verified first, then the expert scoring method is used to enhance the research efficiency of the paper, and then different research indicators are selected to turn them into primary, secondary and tertiary levels, and then they are elaborated. Again, from the perspective of Civic Education, an empirical study was conducted on the communication efficiency of environmental new media. Through the evaluation model established, new media of new environmental types were selected, sample data were collected and processed, and the evaluation model was used to conduct an empirical study on the communication effectiveness of new media. Based on this, combined with the empirical analysis, this paper explores the communication efficiency of environmental news media from the perspective of Civic Education. From the perspective of empirical research, this paper provides a comprehensive assessment of the current communication effect of new media and puts forward relevant countermeasures and suggestions.

The data analysis method of this thesis is mainly through questionnaire surveys of relevant researchers in order to collect and assemble the views of relevant experts. After the indexes were determined, a more systematic and scientific evaluation index system was formed by constructing a judging matrix, distributing questionnaires, inviting relevant experts to score, and further defining the weights of each index. Secondly, the complex multi-objective decision-making problem was regarded as a whole; the target problem was decomposed into multi-objective problems, the target problem was divided into target, criterion level, and operation level, the elements were compared to derive the weights of each element, and, finally, the comprehensive evaluation results were obtained. Finally, search engine technology on the web was used to obtain relevant data and information so as to achieve precise and targeted capture, and then classification was made according to specific rules and guidelines, and finally a database was formed. In this paper, a sample survey method was used to collect information on the specifics of the assessment. The empirical test part of this thesis used an online data collection method to obtain relevant data in a specific time period through web-based data mining techniques, and then tallied the total scores of each assessment account.

### 3.2. Selection of Experts

Hierarchical analysis was used to minimize the error caused by extreme data by counting the score data of 20 experts and deriving different geometric means. Expert authority is the authority of an expert on an issue, and its authority has a great impact on the credibility of the assessment, so it must be measured in a quantitative way. It is expressed by the authority coefficient (Cr) of the expert, which is determined by his or her own assessment results. Cr = (Ca+ Cs)/2 is the expert authority coefficient and Cr > 0.7 is generally considered credible. Table 1 lists the expert authority coefficients for this study, and the results show that the findings of this paper are credible.

### 3.3. Indicator Selection

From the perspective of ideological and political education, the evaluation index system of new media for environmental protection education should be based on a comprehensive understanding of its dimensions and connotation, grasp its characteristics and essence, and select each index as scientifically, objectively, and comprehensively as possible to make it representative and reflect various factors affecting communication efficiency. This paper uses the design ideas and indicators of the current communication effect evaluation indicator system for reference, so that the indicator structure is clear, the weight is clear, and the logic is scientific, which not only reflects the essence of communication efficiency, but also ensures the accuracy and rationality of the evaluation results and follows the scientific and comprehensive principles of indicator design. Table 2 lists the specific indices.

### 3.4. Indicator Description

As shown in Table 3, based on the previous study, this paper divides the formation process of media communication effectiveness into three stages: exposure, perception and attitude. Because the short-term behavior of the audience is difficult to measure and lacks operability, there is no evaluation index for selecting behavior types in this paper. At the same time, the ultimate goal of the indicator system is to use and practice, so that it can conduct a scientific and comprehensive evaluation of the communication effect of environmental information and improve its communication ability and effect. Therefore, the evaluation index system should be based on reality to ensure its practicality and ease of use. The data contained in the indicators should be easy to obtain, and the difficulties in obtaining them are closely related to the operability of the empirical test.

### 3.5. Establishment of Index Weight

Based on the above established evaluation index system of communication efficiency of environmental education new media, Yaahp software is used to give a weighted average of the ranking vector of each expert, and finally the weight of each index in the comprehensive evaluation system is obtained. The weighted distribution of each indicator is listed in Table 4.

### 3.6. Data Sources

This paper selects the ranking list of environmental protection and ecological environment index released by the Qingbo big data platform, selects the WeChat official accounts of each major media environment from the top 30 official accounts, and uses the WCI index of Qingbo big data platform for the latest ranking. Finally, Polaris Environmental Protection, Environmental Protection Hydrosphere, Environment of China, E20 Water Network Solid Waste Network, and Environmental Assessment enthusiast network were selected (see Table 5). WCI indexes were 695.46, 711.58, 883.92, 716.45, and 679.82.

### 3.7. Data Processing

Because these data are obtained directly from official websites and official accounts, as well as from official published content, the dimensions of these data are different. For example, content authenticity, authority, content richness, and other indicators are between 1–5, while the reputation value is tens of thousands, which does not belong to the first level dimension and has no comparability. Therefore, in order to make the scoring results of each official account more reasonable, it can be converted to the initial value minus the minimum value, and then divided by the difference between the maximum value and the minimum value, so as to standardize the index. Table 6 shows the sampling data normalized to the environmental education category of WeChat.

## 4. Empirical Analysis

### 4.1. Evaluation Results

We normalized the collected data and then scored according to the indicator system model established above. We multiplied the score of each indicator by the weight, and finally added the scores of each dimension to obtain the comprehensive score and rankings shown in Table 7.

From the perspective of ideological and political education, the official account represented by E20 Water Network Solid Waste Network has a comprehensive score of 69.06. China’s environment WeChat official account ranked second with a score of 65.26. The third place was “environmental water circle”, with 62.44 points. From the WeChat official account, the EIA enthusiast website ranked fourth, with a comprehensive score of 51.27. Polaris Environmental Protection Network ranked fifth with a score of 33.50.

### 4.2. Analysis of Evaluation Results

#### 4.2.1. Overall Situation Analysis

As shown in the data in Table 8, the overall ranking shows a significant difference from the index screening results based on the WCI index of the ClearBridge platform. On the WCI ranking list from high to low, according to the network communication efficiency evaluation system constructed in this article, after reordering, are E20 Water Network Solid Waste Network, China Environment, Environmental Hydrosphere, Environmental Assessment Lovers Network, and Polaris Environmental Protection Network.

From the score results, the contact efficiency of environmental education official account is the highest, reaching 81.3%. In terms of cognitive efficiency and attitude efficiency, the score rate of the environmental education WeChat official account is not high, 60.7% and 71.5%, respectively. From the perspective of ideological and political education, the environmental protection WeChat official account has a good effect in attracting the attention of college students, which can effectively carry out ideological and political education and improve their ideological awareness. At the cognitive level, as new media bear the responsibility of transmitting environmental protection knowledge and concepts to college students, environmental information is the core of the development of new media for environmental protection, and the content directly affects the cognitive level of college students, fully reflecting the importance of cognitive efficiency in new media for green education. In terms of attitude effectiveness, only one indicator of identity shaping is set, and its score is as high as the full score, but the lowest one is only 4.0. There is a big gap between the two, while the score of attitude efficiency is slightly lacking. This shows that the influence of the official account is not prominent enough in terms of college students’ group identity to influence college students’ emotions and attitudes.

#### 4.2.2. Analysis of Contact Effectiveness Results

Table 9 shows that the scores of legal certification are very high. Except for the EIA enthusiast website, all the others have full marks, because these accounts are officially certified and their personal profiles are relatively complete, which is a direct reflection of the platform’s media reputation. If an account has formal authentication and complete information, college students will feel that the credibility of the account is higher and the quality of information can be guaranteed, leaving a reliable impression on college students, that is, the integrity of personal data. Students can judge whether the account is suitable for them through the content of their personal data, and then decide whether to continue reading. The media influence indexes of different accounts vary greatly. Among them, the scores of Environmental Protection Hydrosphere and E20 Water Network Solid Waste Network are the highest, 9.07 and 9.73, respectively. The scores of China Environment and Polaris Environmental Protection Network are the lowest, especially Polaris Environmental Protection Network at 1.34.

#### 4.2.3. Analysis of Cognitive Efficacy Results

Table 10 shows that in terms of perceived effectiveness, the scores are in two distinct gradients, with China Environment, EIA Enthusiast.com, and E20 Water Solid Waste.com scoring the highest with 54.71, 44.1, and 35.1 points; and Environmental Protection Water Circle scoring the lowest with 21.9 points. The Chinese environment is the official account of the mainstream media, which has a high media credibility and guarantees the authenticity, objectivity, and authority of the news. The lowest score of information output of the Environmental Protection Hydrosphere is due to the fact that most of its articles are original, there are few authoritative information sources, and many professional terms are published, which makes it difficult for non-professionals to understand, resulting in a low score on the information production index of the Environmental Protection Hydrosphere official account. In terms of cultural heritage, Polaris Environmental Protection Network has the lowest score. This is mainly because Polaris Environmental Protection Network focuses on environmental protection projects, enterprises, policies, etc., rarely releases environmental protection knowledge, and rarely publicizes and promotes environmental awareness, so it lags behind in cultural heritage. In addition, the scores of each official account in social monitoring indicators are not much different, all within 10 points. However, the five official accounts generally have insufficient supervision on college students’ behavior, and the relevant contents need to be further improved. From the scores of the coordination and communication indicator table, the EIA enthusiast network scored the highest, 7.19, mainly because it did a good job in providing services for college students and publicizing environmental policies. The scores of China Environment and Polaris Environmental Protection Network are not high, at 3.77 and 4.9, respectively.

#### 4.2.4. Result Analysis of Attitude Effectiveness

Attitude efficacy refers to the influence of people’s thoughts or values on emotions, and it is the behavioral orientation and willingness to express information after recognition. In the evaluation indicators established in this study, identification modeling includes “college students’ participation” and “college students’ sense of identity”. Table 11 shows the score results of the attitude effectiveness index.

From the perspective of attitude efficiency, the attitude efficiency of the Environmental Protection Hydrosphere official account shows its most obvious advantage of 36.86 points, which occupies an absolute advantage in college students’ participation and identity. At the same time, this official account also has the highest number of likes, with an average of 12 likes for each article. Among the five official accounts, only one account has 10 likes, which makes college students more recognized on this topic. In contrast, the attitude efficiency index of Polaris Environmental Protection Network and EIA hobby network fell behind significantly, being 3.78 and 6.35, respectively. In these data, the average number of hits of articles on the EIA enthusiast website is 2142, while the average number of likes is only two. The average number of clicks on Polaris is only 1788, and the average number of likes is only three, indicating that college students’ attention and recognition are relatively low.

## 5. Conclusions

This paper discusses the importance of ecological civilization education in today’s social environment from the perspective of communication efficiency of new media of environmental protection education and analyzes the current communication situation of new media of environmental protection education. Based on the theory of communication effect and the theory of media ideological and political education, this paper organically combines the two and establishes the evaluation index system of new media for environmental protection education based on the theory of communication effect of “ecological civilization ideological and political education”. Through sorting and screening the evaluation indicators of relevant literature and new media, the relevant indicators were determined. Using the Delphi method and the analytic hierarchy process, experts ranked and rated the importance of each indicator, determined its weight according to the scoring results, and built an evaluation system. On this basis, an empirical study was carried out.

In this paper, we took environmental education as an example and selected five most representative public numbers for empirical analysis based on Qingbo’s big data and rated them. At the same time, the index was compared with the WeChat Promotion Index to verify the operability of the index system. This study found that the communication efficiency of new media has a positive impact on environmental awareness education.

In terms of contact efficiency, the higher the willingness of college students to contact the media, the more significant the educational effect reflected, and the more significant the cognitive efficiency and attitudinal efficiency. Therefore, universities and the government should increase the ideological and political education on environmental protection for college students, enhance the publicity effect of environmental protection new media, make the information content of new media richer, and let college students actively participate in this activity, so as to further promote the ecological civilization awareness of college students and promote the sustainable development of ecological environment.

In terms of cognitive efficiency, because environmental issues are more specialized and knowledge-dependent, and environmental reports often involve economic, scientific, technological, biological, and other fields, it is more difficult to understand them, and the audience threshold for complex and specialized environmental issues is higher. Environmental issues form an “elite-elite” communication pattern, and the audience of environmental media is almost limited to government organizations, environmental practitioners, members of environmental public interest organizations, experts and scholars, and other related groups, which plays an important role in improving the communication efficiency of new media.

In terms of attitudinal effectiveness, according to the evaluation of the environmental protection public number, it is found that the number of likes on the environmental protection WeChat public number is not high, and some accounts do not even have single-digit likes. This is partly because the general public has not yet developed the habit of “liking”, and partly because the content is boring, obscure, and not interesting to a narrow audience, which over time reduces user acceptance. Moreover, due to the special nature of environmental information, it is difficult for the general audience to understand the professional rankings and speeches, and even if they want to express their own views, they can only stay on the surface and cannot discuss deeply.

## 6. Suggestions

### 6.1. Focus on the Content Orientation and Originality of Environmental Education Publicity

With the rapid development of the network, people obtain more information in more flexible ways. A large amount of copied and reprinted content not only does not have any innovation, but will cause the loss of users. On the WeChat public platform, the production of environmental education content that meets the needs of the audience and is close to the life of the audience is an issue that all participants in environmental education should pay attention to. For example, in the current official account of “environmental education”, both the government and businesses are based at the city level, or even the community. For example, the main body of the communication platform of ecological environmental education in Shinan District of Qingdao is the Shinan District Government of Qingdao; Ningbo Dark Blue Environmental Education Consulting Co., Ltd. is the main communication body of Ningbo Dark Blue Environmental Education Service Center. In the process of publicizing environmental education, new media should pay attention to a small part of environmental education policies, services, and practices, and be closer to the lives of local residents.

### 6.2. Open the Feedback Channel and Provide A Platform for the Audience to Participate in the Discussion

From the perspective of the audience, the new media of environmental protection education is the audience of environmental problems and the bearer of environmental crisis, so it is called the “negative voice”. In environmental communication, people often appear as victims. Public opinion generally has a lack of rationality, strong subjective consciousness, and corresponding expertise, so it is difficult to put forward more reasonable and professional opinions. Therefore, in most cases, new media plays the role of an information transmitter in environmental communication, but in fact, the public is not really involved in environmental issues, let alone participating in decision making. The audience has no voice and no feedback, which makes it difficult to improve the degree of participation. Therefore, the new media platform must expand the audience’s feedback channels, improve its interaction mechanism, and strengthen the communication with users. The new media platform for environmental protection education should strengthen the communication and interaction with the audience. Whether leaving messages in articles or sending private messages in the background, there should be a good feedback channel to actively respond to the audience’s opinions, answer the audience’s questions, and conduct valuable and rational discussions below to stimulate the public’s enthusiasm for environmental issues. Only by making people feel their voice will they be more willing to express their views, so as to improve their communication effect.

### 6.3. Give Play to the Role of Cultural Heritage and Improve People’s Awareness of Environmental Protection

The construction and development of an ecological civilization cannot be separated from the participation of the people, and it is more important to strengthen citizens’ awareness of ecological civilization. Therefore, the new media of environmental protection education should give full play to its role in cultural inheritance, based on its own position, pay more attention to the content, increase the publicity of environmental protection knowledge and technology, strengthen the education of environmental protection, advocate green spirit, gather green forces, and establish a good atmosphere of green civilization. China has very rich ecological and cultural resources. Many ecological cultures have evolved from ancient ecological civilization, passed down from generation to generation, and carry the precious experience and wisdom of mankind. At present, the new media focus on the inheritance of ecological culture, pay attention to the use of society, lack of human resources support, and cannot promote social environmental protection. Therefore, the new media of environmental protection education should give full play to the role of education and inheritance of traditional ecological culture, tell the ecological culture story better, let the public understand and inherit the ecological culture, make the ecological culture truly integrated into people’s hearts, and cultivate the ecological ethics consciousness. The people with good ecological aesthetic consciousness have promoted the formation of the whole social civilization.

### 6.4. Publicize Environmental Education Knowledge and Create a Good Public Opinion Environment

Environmental events, environmental knowledge, environmental information, and other forms form the public’s understanding of the environment, create a green public opinion environment, and thus promote the harmonious development of society. The transformation of the media has transformed China’s environmental protection publicity from “media oriented environmental news” to “media and mass oriented environmental protection publicity”, and finally formed an environmental protection public opinion field with the participation of the whole people. In the current environmental situation, the publicity of environmental education knowledge should pay more attention to the information exchange between the media and the public. The media guides and cultivates environmental public opinion through the publicity of environmental education, while the public corrects and maintains environmental public opinion through their own publicity and public opinion supervision. The two are interdependent and affect each other. Today’s public is very independent, and they can provide knowledge of environmental protection through many ways. For this problem, in the process of spreading environmental knowledge, communicators should not only strengthen their professional quality, but also pay attention to conveying the information that the public needs and can resonate with the public.

## Figures and Tables

**Table 1:** Expert authority coefficient.

Round	Familiarity	Judgment Basis	Authority Coefficient
First round	0.827	0.864	0.846
Second round	0.826	0.895	0.861

**Table 2:** Evaluation Index Framework of New Media for Environmental Protection Education from the Perspective of Ideological and Political Education.

Level I Indicators	Secondary Indicators	Third Level Indicators
Contact efficiency	Legal operation	Platform certification
		Complete data
	Media impact	Popularity
		Activity
Cognitive efficacy	Information production	Information authenticity
		Information authority
		Originality of information
		Information readability
		Content richness
	Cultural heritage	Popularization of knowledge and skills
		Ecological culture communication
	Social environment supervision	Supervise government actions
		Supervise school behavior
		Supervise student behavior
	Coordination and communication	Dissemination of environmental protection policies
		Smooth feedback channels
		Provision of public services
Attitudinal efficacy	Identification shaping	Participation of college students
		College students’ identification

**Table 3:** Definition of Evaluation Indicators.

Evaluating Indicator	Description of Meaning
Platform certification	Authenticity and authority of the account, that is, whether the account has been authenticated
Complete data	Completeness of profiles and other materials that college students can see
Popularity	Number of followers of the account
Activity	Sending time and frequency of account
Information authenticity	Authenticity of measured sample content
Information authority	Whether to quote authoritative sources
Originality of information	Whether the measurement sample is original
Information readability	Whether the content is vivid, popular, easy to understand, and interesting
Content richness	Measure whether the content of the sample is distributed in various forms, such as video, audio, pictures, and other multimedia applications
Popularization of knowledge and skills	Popularization of environmental protection skills of measurement objects
Ecological culture communication	Status of the measurement object on the transmission of ecological culture
Supervise government actions	Measure whether there is any content in the sample to supervise the government’s behavior
Supervise school behavior	Whether there is content in the measurement sample to supervise the behavior of colleges and universities
Supervise student behavior	Measure whether there is content in the sample to supervise students’ behavior
Dissemination of environmental protection policies	Coordination of the tripartite relationship among government, universities, and students in the survey objects
Smooth feedback channels	Measure the response to comments and messages in the sample and the quality of comments and messages
Provision of public services	Whether the measurement object provides college students with some service channels, link guidance, etc
Participation of college students	Average reading index
College students’ identification	Average like index

**Table 4:** Weights of evaluation indicators for communication efficiency of environmental protection education new media from the perspective of ideological and political education.

Level I Indicators	Weight Value	Secondary Indicators	Weight Value	Third Level Indicators	Weight Value
Contact efficiency	0.2370	Legal operation	0.5261	Platform certification	0.6247
				Complete data	0.3753
		Media impact	0.4739	Popularity	0.4349
				Activity	0.5651
Cognitive efficacy	0.5066	Information production	0.2557	Information authenticity	0.2895
				Information authority	0.1989
				Originality of information	0.1702
				Information readability	0.2079
				Content richness	0.1335
		Cultural heritage	0.2168	Popularization of knowledge and skills	0.5559
				Ecological culture communication	0.4441
		Social environment supervision	0.2833	Supervise government actions	0.3572
				Supervise school behavior	0.3805
				Supervise student behavior	0.2623
		Coordination and communication	0.2442	Dissemination of environmental protection policies	0.3472
				Smooth feedback channels	0.3679
				Provision of public services	0.2849
Attitudinal efficacy	0.2564	Identification shaping	1.00	Participation of college students	0.4871
				College students’ identification	0.5129

**Table 5:** Weekly list of WeChat WCI index of environmental education in the top five accounts.

Official Account	Release	Read	Headlines	Average	Likes	WCI
Environment of China	40/58	242,098	191,119	5162	1283	883.92
E20 Water Network Solid Waste Network	18/24	62,922	46,404	4850	700	719.45
Environmental Protection Hydrosphere	17/33	86,478	45,476	3941	289	711.58
Polaris Environmental Protection Network	18/30	72,089	44,591	3805	192	695.46
EIA enthusiast website	15/25	71,821	35,667	5140	159	679.82

**Table 6:** Scores of each index after standardization.

Third Level Indicators	Polaris Environmental Protection Network	Environmental Protection Hydrosphere	Environment of China	E20 Water Network Solid Waste Network	EIA Enthusiast Website
Platform certification	100	100	100	100	100
Complete data	100	100	100	100	0
Popularity	0	100	4.0	89	94
Activity	4.0	0	100	43	43
Information authenticity	69	0	100	81	100
Information authority	97	0	100	80	92
Originality of information	26	100	50	41	0
Information readability	0	46	100	50	62
Content richness	40	100	99	96	0
Popularization of knowledge and skills	0	100	5.7	45	53
Ecological culture communication	0	86	100	76	25
Supervise government actions	55	0	100	70	53
Supervise school behavior	100	0	47	81	82
Supervise student behavior	23	0	91	50	100
Dissemination of environmental protection policies	57	0	78	80	100
Smooth feedback channels	0	100	37	66	71
Provision of public services	76	43	0	51	100
Participation of college students	0	100	29	50	51
College students’ identification	31	100	71	61	0

**Table 7:** Overall score and ranking of official account communication efficiency based on ideological and political education.

	Contact Efficiency (0–24.8)	Cognitive Efficacy (0–50.77)	Attitudinal Efficacy (0–25.75)	Total Score (0–100)	Ranking (1–5)
Polaris Environmental Protection Network	11.27	19.78	2.67	33.50	5
Environmental Protection Hydrosphere	19.66	17.25	25.75	62.44	3
Environment of China	15.57	29.94	19.97	65.26	2
E20 Water Network Solid Waste Network	19.10	27.25	22.93	69.06	1
EIA enthusiast website	16.92	29.33	5.24	51.27	4

**Table 8:** Score rate of primary indicators.

Level I Indicators	Full Score	Highest Score	Minimum Score	Average	Score Rate
Contact efficiency	35.9	30.77	22.38	27.61	81.3%
Cognitive efficacy	61.88	41.05	28.36	36.28	60.7%
Attitudinal efficacy	36.86	36.86	5.1	26.44	71.5%

**Table 9:** Score and Ranking of Contact Effectiveness.

Contact Efficiency	Legal Certification (Two Level Weighting)	Media Impact (Two Level Weighting)	Contact Efficiency (First Level Weighting)	Ranking
Environmental Protection Hydrosphere	22.26	9.73	30.77	1
E20 Water Network Solid Waste Network	22.26	9.07	30.22	2
EIA enthusiast website	9.7	10.53	28.03	3
Environment of China	22.26	5.64	26.68	4
Polaris Environmental Protection Network	22.26	1.34	22.38	5
Full score	22.26	13.88	35.9	

**Table 10:** Cognitive Efficacy Scores and Ranking.

Cognitive Efficacy	Information Production (Two Level Weighting)	Cultural Heritage (Two Level Weighting)	Social Supervision (Two Level Weighting)	Coordination and Communication (Two Level Weighting)	Total Cognitive Score	Ranking
Environment of China	25.34	5.97	19.63	3.77	54.71	1
EIA enthusiast website	21.26	4.14	11.51	7.19	44.1	2
E20 Water Network Solid Waste Network	10.93	5.78	9.80	5.59	35.1	3
Polaris Environmental Protection Network	8.53	1.1	10.88	4.9	25.41	4
Environmental Protection Hydrosphere	5.27	9.88	1.1	5.65	21.9	5
Full score	26.79	10.90	29.01	8.84	60.77	

**Table 11:** Score and Ranking of Attitude Effectiveness.

Attitudinal Efficacy	Identification Shaping (Two Level Weighting)	Participation of College Students (Three Level Weighting)	College Students’ Identification (Three Level Weighting)	Ranking
Environmental Protection Hydrosphere	36.86	23.93	24.15	1
E20 Water Network Solid Waste Network	34.04	21.3	28.91	2
Environment of China	31.08	5.84	26.46	3
EIA enthusiast website	6.35	6.35	1.1	4
Polaris Environmental Protection Network	3.78	1.1	3.78	5
Full score	36.86	23.93	24.15	

## Data Availability

The data are not publicly available due to privacy restrictions.
